# A Pilot Study on Cortical Auditory Evoked Potentials in Children: Aided CAEPs Reflect Improved High-Frequency Audibility with Frequency Compression Hearing Aid Technology

**DOI:** 10.1155/2012/982894

**Published:** 2012-10-31

**Authors:** Danielle Glista, Vijayalakshmi Easwar, David W. Purcell, Susan Scollie

**Affiliations:** ^1^National Centre for Audiology, Faculty of Health Sciences, The University of Western Ontario, 1201 Western Road, Elborn College, Room 2262, London, ON, Canada N6G 1H1; ^2^National Centre for Audiology and Program in Health and Rehabilitation Sciences (Hearing Sciences), Faculty of Health Sciences, Western University, London ON, Canada N6G 1H1; ^3^School of Communication Sciences and Disorders and National Centre for Audiology, Faculty of Health Sciences, Western University, London ON, Canada N6G 1H1

## Abstract

*Background*. This study investigated whether cortical auditory evoked potentials (CAEPs) could reliably be recorded and interpreted using clinical testing equipment, to assess the effects of hearing aid technology on the CAEP. *Methods*. Fifteen normal hearing (NH) and five hearing impaired (HI) children were included in the study. NH children were tested unaided; HI children were tested while wearing hearing aids. CAEPs were evoked with tone bursts presented at a suprathreshold level. Presence/absence of CAEPs was established based on agreement between two independent raters. *Results*. Present waveforms were interpreted for most NH listeners and all HI listeners, when stimuli were measured to be at an audible level. The younger NH children were found to have significantly different waveform morphology, compared to the older children, with grand averaged waveforms differing in the later part of the time window (the N2 response). Results suggest that in some children, frequency compression hearing aid processing improved audibility of specific frequencies, leading to increased rates of detectable cortical responses in HI children. *Conclusions*. These findings provide support for the use of CAEPs in measuring hearing aid benefit. Further research is needed to validate aided results across a larger group of HI participants and with speech-based stimuli.

## 1. Introduction

A growing body of literature exists on the use of cortical auditory evoked potentials (CAEPs) in assessing neural activity in NH and HI listeners. Such measures reflect the sum of synchronous, time-locked neural activity detected at the level of the central auditory system, related to the strength (amplitude) and timing (latency) of a response [1, 2]. For these reasons, CAEPs have been suggested for clinical use in monitoring changes in neural activity associated with auditory rehabilitation (e.g., hearing aids). The P1-N1-P2 complex is one type of evoked potential, comprised of slow components ranging from 50 to 300 msec in latency [2]. The peaks of the complex are thought to reflect neural activation of the central auditory system in response to the spectral and temporal properties of a given stimulus [3, 4]. Studies including normal hearing (NH) and hearing impaired (HI) participants conclude that CAEPs characterized the P1-N1-P2 complex, elicited by tonal stimuli and various speech tokens, can be reliably recorded to produce distinct neural patterns in both aided and unaided conditions [3, 5–7]. Such literature is mainly comprised of studies including research-grade equipment using multiple testing channels (greater than two). Few studies have looked at using CAEPs with commercially available clinical testing equipment (e.g., Hear lab). Commercially available systems are commonly comprised of a single-channel recording system [8, 9]; such equipment has been proposed for use in aided assessment to evaluate infant hearing aid fitting using CAEPs [10]. Literature suggests that aided CAEPs may be able to provide information related to neural detection and audibility of aided and unaided sound [7, 8]. Further research is needed to help quantify the relationship between CAEPs and hearing aid benefit. 

When sound is processed through a hearing aid, it is necessary to understand what the hearing aid is doing to the signal. For this reason, recent research in this area has focused on amplification-related modifications to the CAEP stimulus, related to factors such as poor signal-to-noise ratio (SNR) and/or rise time [11–13]; both of which may interact with the input stimulus level used in the fitting process. Billings and colleagues (2011) have investigated whether hearing aids modify stimulus characteristics such as SNR, suggesting that these changes can affect the CAEP in a way that does not reliably reflect hearing aid gain [11, 14]. Low stimulus levels were used to avoid loud hearing aid output levels with NH listeners [11]. Such stimulus levels are atypical in studies of hearing aid validation where supra-threshold levels representing conversational levels of speech (e.g., 55–75 dB SPL) are traditionally used [15]. Because the stimulus level can interact with nonlinear signal processing in hearing aids, a lower level stimulus will receive more gain than higher level stimuli. For this reason, consideration of stimulus level is important if the goal of CAEP measurement is to characterize the aided response to sound. The effect of SNR has been investigated in NH listeners only and hence bound to vary with HI listeners as the effect of SNR is largely dependent on the relative level of hearing aid internal noise and hearing thresholds. In a recent study by Easwar et al. [16], the stimulus onset altering effect of a hearing aid on CAEPs elicited with tone burst stimuli of varying rise times was investigated. Findings suggest that alterations in the tone burst stimuli caused by hearing aid processing are not large enough to significantly influence CAEP responses in NH listeners with hearing aid processed stimuli [16]. Further research is needed to validate findings with HI listeners, fitted with varying types of clinically common hearing aids. In general, these studies alert clinicians and researchers of the potential for hearing aid signal processing to interact with CAEP stimuli, in ways that may or may not affect the response. More research is needed in this area to understand fully whether CAEP methodologies are sensitive to other aspects of hearing aid signal processing, for several reasons. One reason may be to investigate whether signal processing acts as a confound: does it alter the CAEP stimulus in an unexpected manner, as has been investigated elsewhere [12, 13, 17]. Another reason may be to pursue whether CAEP changes correlate with behavioral changes, specifically for hearing aid signal processing that causes performance improvement or decrement. This paper will mainly consider the latter type of question.

One example of a change in hearing aid signal processing is the application of frequency lowering. Studies suggest that frequency lowering hearing aid technology may benefit adults and children with high-frequency hearing loss (refer to [17] for a summary). For the purpose of this paper, change in audibility due to frequency lowering in the form of nonlinear frequency compression (NLFC) was investigated using CAEPs. In general, NLFC splits the incoming hearing aid signal into two channels. The high-frequency channel is compressed into a narrower bandwidth. This results in sound being lowered in frequency within the high-frequency channel only [18]. Research suggests that NLFC hearing aid processing can provide speech sound detection perception benefit for some adults and children with high-frequency hearing loss and that benefit varies across individuals [18–23]. Specifically, listeners with severe-to-profound high-frequency hearing loss are most likely to derive large benefits, because the technology changes audibility of high-frequency cues. Therefore, the aided CAEP may be sensitive to the increased audibility produced by NLFC hearing aids. However, no studies have investigated whether aided CAEPs might change in response to NLFC activation. 

The present study investigated the use of CAEP measures to evaluate audibility of tone bursts with and without NLFC frequency lowering, using hearing aid-processed stimuli with HI listeners, in comparison to a group of NH listeners. Since the end goal of using aided CAEPs is to evaluate the benefit of hearing aid fittings clinically, this study uses commonly available clinical equipment for recording CAEPs. Research grade equipment with multiple channels including a channel to detect eye blink, although may provide higher quality data with additional information such as scalp topography, may be difficult to use clinically due to time, cost, and feasibility concerns. Modifications to clinically available testing equipment, the Bio-logic Navigator Pro System, allowed tone burst stimuli to be presented directly to the direct audio input (DAI) of a hearing aid with an audioshoe connector in aided testing conditions. The purpose of this study was to investigate the following: (1) whether CAEPs could be reliably recorded and interpreted using Bio-logic Navigator Pro testing equipment in NH and HI children, and (2) whether CAEPs elicited by high-frequency tone burst stimuli reflected the change in high frequency audibility due to use of NLFC hearing aid technology. 

## 2. Materials and Methods

### 2.1. Participants

Participants included 15 NH children (mean = 14.4 years, range = 11–18 years) and 5 HI children with high-frequency hearing loss (one 11-year old, one 14-year old, and three 18-year olds). Children in this age range were chosen for this pilot study as they are likely to understand and follow instructions during testing (e.g., keep awake during recording) and their CAEPs may be considered representative of a maturing system [24]. NH children were included to provide a reference of typically maturing CAEPs without the influence of hearing impairment. The HI children were recruited from a study of acclimatization to frequency lowering hearing aids [25], in which they were provided with study hearing aid for approximately four months in duration. In the present study, data from one HI child were excluded because of unexplained acoustic artifacts in the hearing aid output measured with study-specific stimuli. Pure-tone air conduction testing was carried out in a double-walled sound booth using Etymotic Research ER-3A insert earphones across octave and interoctave audiometric frequencies between 0.25 and 8 kHz (GSI-61 Audiometer; [26]). Routine otoscopic examination ruled out any contraindications such as active discharge, occluding wax, or foreign body in the ear canal. Participants reported no significant history of neurological or otological problems. The study protocol was approved by the University of Western Ontario Health Sciences Research Ethics Board. 

The eligibility criteria for the NH participants included passing a hearing screen at 15 dB HL. For the HI participants, hearing thresholds were measured by coupling the insert earphones to each participant's personal earmolds. Eligibility criteria included bilateral sensorineural hearing impairment, sloping to at least a moderately severe high-frequency pure-tone average (HF-PTA) hearing level averaged across 2, 3, and 4 kHz. Hearing threshold data is displayed for each case in the Results section. Participants were required to be full-time users of digital behind-the-ear (BTE) hearing aids prior to entering the study and to maintain full time use of the study hearing aids prior to CAEP measurement. Four of the participants were binaural hearing aid wearers with symmetrical high-frequency hearing loss within 10 dB, based on HF-PTA. One participant with an asymmetrical hearing loss was monaurally aided in the better ear (Case 5); a hearing aid trial on the ear with a greater level of impairment (i.e., a profound hearing loss) was not successful. Hearing thresholds were measured at the beginning and end of the study. All participants demonstrated hearing levels within 10 dB of baseline over the course of the study. The presence of cochlear dead regions was assessed using the TEN test in dB HL [27].

### 2.2. Stimuli

Tone bursts at 2 and 4 kHz were generated from the Bio-logic Navigator Pro (v7.0.0). Using stimulus parameter options available within the Bio-logic software, cycle numbers were held constant across stimuli with a rise/fall Blackman ramp of 20 cycles each and a plateau of 80 cycles. This produced a 40 msec plateau and 10 msec rise/fall time for the 2 kHz tone burst and a 20 msec plateau and 5 msec rise/fall time for the 4 kHz tone burst. 

### 2.3. Testing Conditions

Testing was carried out in sessions no longer than two hours. One session was required for NH participants and two were required for HI participants. Breaks were given when requested. Participants were seated watching a muted movie of their choice with active subtitles and were instructed to ignore the stimuli played [28, 29]. The sequence of stimulus presentation in a session was randomized. For the NH participants, test ear was alternated across participant order numbers. Unaided monaural testing was completed by routing the stimuli directly from the Navigator Pro to Bio-logic broadband insert earphones coupled to foam tips. 

For the HI participants, aided monaural testing was carried out for the better listening ear, according to pure-tone average (PTA). Stimuli were routed directly from the Navigator Pro (and through an audio isolation transformer) to the DAI of the hearing aid with an audioshoe connector coupled to a study worn hearing aid, using a custom cable [30]. This DAI strategy was chosen to remove the effects of room acoustics, head azimuth/movement, and listener distance from a loudspeaker that could have affected the accuracy and reliability of sound-field presentation of stimuli. The stimulus presentation level strategy described below was chosen to present the unaided stimuli to the NH participants at a dial level of 70 and to provide individualized amplification to each HI participant for the same input level using the DAI routing. The DAI routing was verified as acoustically transparent (within 2 dB) by routing the test signals through a study hearing aid programmed to have no amplification, through a 2cc coupler to a Type I sound level meter (Larson-Davis 824). The sections below outline the procedures used to derive individual hearing aid fittings and details of stimuli and calibration.

#### 2.3.1. Individualized Hearing Aid Fittings

Device fitting followed protocols from the Desired Sensation Level (DSL) method version 5.0 [31, 32] as implemented within the Audioscan Verifit VF-1 (a clinical test system used for hearing aid analysis). Each participant was fitted with Phonak Naida IX SP BTE hearing aids. Hearing devices were worn for the entire duration of the study with the gain/advanced features held constant throughout. Volume control, digital noise reduction, and automatic program selector features were disabled. Prescriptive targets were matched using simulated real ear measures incorporating individual real ear to coupler difference values. We selected a coupler-based verification strategy to reduce room noise/reverberation effects and concerns with feedback during verification; this promoted test environment consistency and replicable measures across the repeated fitting appointments. Aided test box measurements of speech at 55, 65, 70, and 75 dB SPL and for a 90 dB SPL pure-tone signal were completed during fitting appointments. Hearing aids were adjusted to provide the best possible match to targets. NLFC settings (i.e., cut-off frequency and compression ration) were individualized according to established procedures [30, 33] using manufacturer specific fitting software (Case specific settings are provided in Section 3 (Figures 3 to 7)).

Aided CAEP testing was completed on two different testing sessions: the first with NLFC enabled (treatment condition) and the second with NLFC disabled (no-NLFC). On average, the NFLC testing session was completed after 15 weeks of acclimatization to NLFC processing (range: 14 to 17 weeks). The no-NLFC testing session was completed 4 weeks after finishing the treatment condition of the study (range: 1 to 7 weeks). Participants were naïve to all details pertaining to the study design. Such details, along with the individualized results, were disclosed to the participants upon completion of the study. 

The potential for hearing aid-induced delay affecting latency values in HI testing conditions was measured using an anechoic box (B&K 4232). Stimuli were presented by playing 2 and 4 kHz tone burst stimuli from SpectraPlus software via a study hearing aid connected to the coupler. The output of the coupler and reference channels of the recordings were compared to each other using a 75 dB SPL input level to estimate the presence of any hearing aid-induced delay. The difference in the onset of stimuli in each recording channel was calculated to be 6.7 msec, on average, across stimuli and testing conditions. Delay values ranged from 6.3 to 7 msec. Due to the insignificance of the calculated delay values compared to the latency of CAEPs, no corrections were applied for the purpose of comparing HI and NH CAEP data.

### 2.4. Presentation Level

A suprathreshold presentation level was determined by generating a tone burst stimulus from the Navigator Pro at a testing dial level of 70 dB in the anechoic test box. The output was measured via broadband insert earphones connected to an HA-2 (25 mm tubing) 2cc coupler and microphone (B&K 4192). SpectraPlus software was used to capture the output RMS of the coupler in dB SPL across the tone burst plateau. The chosen dial level produced presentation levels of 69 and 71 dB SPL (re: 2cc coupler) for the 2 and 4 kHz tone burst stimuli, respectively. 

The same dial level was used for aided testing with HI participants. Additional electroacoustic verification measures of aided stimuli were completed using the same set-up as described previously. Electroacoustic measures allowed individualized estimation of audibility of the tone bursts for each of the hearing aid fittings and testing conditions. Navigator Pro tone bursts generated at the chosen dial level were routed through the study hearing aid set-up, programmed with individualized fittings. Overall rms of each tone burst (including rise, plateau, and fall time) was measured for individual fittings with and without NLFC for each stimulus. Since these measures were made in a 2cc coupler, audiometric thresholds were transformed to SPL in a 2cc coupler using individualized ear canal transforms [31], and the sensation level of each stimulus was computed as aided RMS level minus audiometric threshold, with all values in coupler SPL. To account for the shorter duration of the tone bursts during estimation of tone burst SL values, correction factors of 3 and 6 dB at 2 and 4 kHz, respectively, were subtracted from the SL value obtained for pure tones used during audiometry [34]. A summary of these results along with those for all corresponding CAEP measures is presented in the results section (Table 1). 

### 2.5. Set-Up of CAEP Equipment

An ipsilateral recording (Vertex to ipsilateral mastoid with ground Fpz) was obtained using the Navigator Pro. Tone bursts were presented at the rate of 0.5 stimuli/sec. This interstimulusinterval was the same as that used to acoustically record in the aided condition. Each recorded electroencephalogram (EEG) sweep included 100 msec of prestimulus baseline (relative to tone burst onset) and 966 msec of post stimulus activity. EEG was amplified 50000 times and digitized at the rate of 480.03 Hz. Responses were bandpass filtered between 0.1 and 100 Hz online. The artifact rejection threshold was set to ±100 *μ*V. Two averages of 100 sweeps each were obtained for each stimulus condition. Due to study-specific modifications to the traditional testing parameters used in the Bio-logic software, including epoch time/stimulus rate, it was not possible to include a calculation of residual noise level as a stop criterion.

### 2.6. Waveform Interpretation

Data extraction from the Bio-logic Software was limited to averaged data; therefore, previously reported statistical techniques [35] could not be applied to this data set. Averaged CEAP waveforms were exported from the testing equipment and postprocessed using a MATLAB script version 2008b (Mathworks, 2008) including a second order bandpass Butterworth filter (1–15 Hz). Replicated CAEP waveforms for all participants and across testing sessions were then interpreted by two experienced raters using subjective response detection techniques. Data between 0 and 400 msec after stimulus onset were included in rater interpretations [36]; this included a time window spanning beyond that of a traditional P1-N1-P2-N2 complex [2, 37]. 

Research on the maturational effects associated with the CAEP response suggests that changes to the waveform morphology associated with the P1-N1-P2 complex can be observed between the ages of 6 and 18 years [2, 24]. Therefore, based on the age range assessed in this study (11–18 years), the authors chose to interpret the data according to the presence/absence of one or more peaks, rather than using peak picking according to traditional latency values associated with specific responses in the P1-N1-P2 complex. The decision regarding response presence or absence required the agreement of both raters; disagreement resulted in a rating of “absent” for the CAEP response in question. Each rater was blind to the test condition and to the other rater's judgments during interpretation. For a response to be considered present the raters had to agree that at least one peak (according to replicable data) resided within the chosen time window. Waveform interpretation results were used in group level analyses for the NH group and in single-subject analyses for the HI cases. In single-subject designs, each participant serves as his or her own control allowing for the opportunity to measure significant changes in performance at the individual level [38], in this study, changes as a consequence of enabling frequency compression. This type of analysis was of particular interest in this study given the varying hearing loss degrees/configurations present in the HI cases, which required individualized frequency compression settings. 

## 3. Results

### 3.1. Interrater Agreement

An interrater reliability analysis using the Kappa statistic was performed using SPSS software to examine consistency among the two raters. Analyses were performed including waveforms from all participants in both conditions. The interrater reliability analysis for the examiners, across all stimuli and conditions, was found to be Kappa = 1 (*P* < 0.001), 95% CI (1, 1), with a standard error of zero. A Kappa value of one implies perfect agreement between the two raters.

### 3.2. Objective Index of Replicability of CAEP

**Figure 1:**
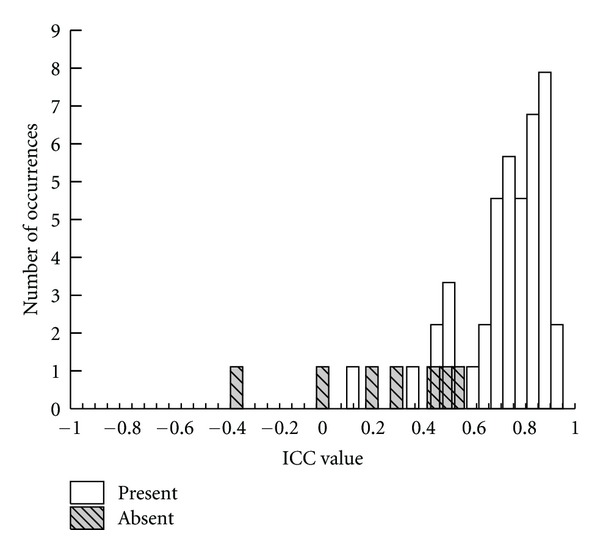
Histogram of ICC values for CAEPs waveforms judged to be present and absent.

The intraclass class correlation coefficient (ICC) has recently been used to quantify similarity between two waveforms [36, 39]. Visual inspection of waveform similarity and corresponding ICC values between two CAEP waveforms have been shown to agree at the group level [40]. The ICC value can provide a measure of the similarity of the waveform amplitude and shape, with higher values representing greater similarity between evoked responses [36, 40]. To investigate the use of ICC measures in waveform interpretation, the present study explored the relationship between ICC values computed for repetition 1 and repetition 2 of the recorded waveforms and final subjective interpretation of presence versus absence of the CAEP. For this purpose, one-way random ICCs were computed between two averages (repetition 1 and repetition 2) for each stimulus condition, for each hearing aid condition. The same time window used for subjective waveform interpretation was used when computing ICC values. Since the distribution of ICC values does not follow a normal distribution, median values are reported across all stimuli and conditions, and 95% confidence intervals (CI) have been limited to the range of the data in the following summary. The median ICC for CAEPs that were judged to be present subjectively was 0.816 (range: 0.11 to 0.97; CI = 0.27–0.96) and median ICC for CAEPs that were judged to be absent subjectively was much lower, 0.278 (range: −0.3 to 0.5; CI = −0.42–0.5). ICC values above .75 are indicative of good reliability [41]. A histogram of ICC values for CAEP waveforms subjectively judged as present and absent is illustrated in Figure 1. 

### 3.3. CAEP Responses with NH Listeners

**Figure 2:**
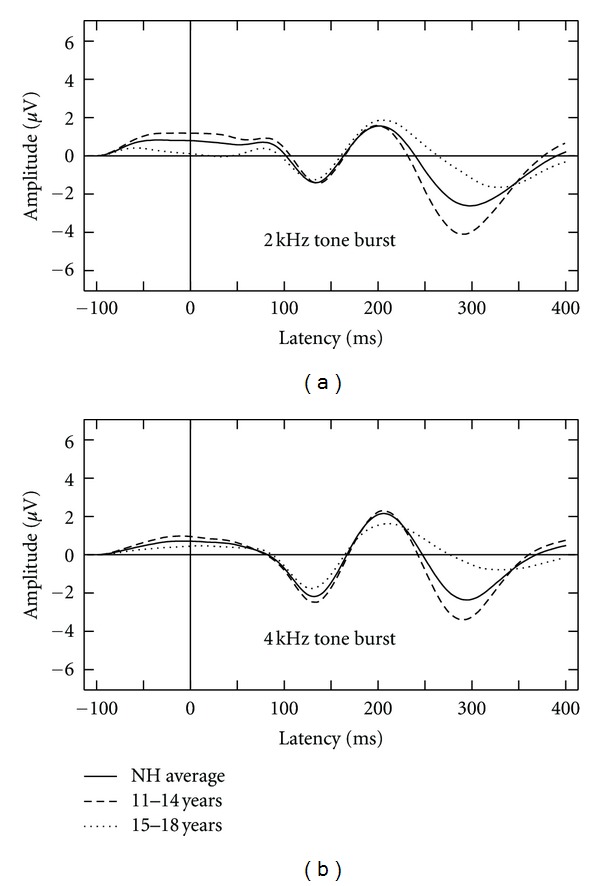
Averaged waveforms displayed according to stimulus for all NH groups including an average of all participants (solid line), those between the ages of 11 to 14 years (dashed line) and those between the ages of 15 to 18 years (dotted line).

Data collected with NH listeners is displayed at the group level according to grand mean waveforms per stimulus (Figure 2), as well as in age separated groups (11–14 and 15–18 years). Of the 15 NH children tested, CAEP responses were judged to be present for most listeners. Absent waveforms were measured for one listener with the 4 kHz tone burst and for two listeners for the 2 kHz tone burst. Related analyses and figures include data from present responses only. 

Developmental changes in the CAEP waveform have previously been reported over the age range that we studied [2, 24]. The effects of age subgroup on the observed CAEP for the NH children were submitted to a two-way analysis of variance (ANOVA) with age group (younger versus older children) as a between-subjects factor and the time window used in CAEP measurement (averaged sample points within each bin) as a repeated measures factor with 8 levels. Chosen bins corresponded to eight 50 msec intervals within the 400 msec window used in this study, equaling that used in previous evaluation of CAEPs [35, 42]. The amplitude of the CAEP waveforms elicited to the 2 kHz tone burst varied as little as 0.078 *μ*V in the 4th bin (corresponding to latency region of 150−199 ms) to as large as 3.28 *μ*V in the 6th bin (corresponding to latency region of 250–299 ms). On average, the 6th bin measured 0.38 *μ*V in the older group compared to −2.9 *μ*V in the younger group. The amplitude of the CAEP waveforms elicited to the 4 kHz tone burst varied as little as 0.003 *μ*V in the 5th bin (corresponding to latency region of 200−249 ms) to as large as 3.03 *μ*V in the 6th bin (corresponding to latency region of 250–299 ms). On average, the 6th bin measured 0.44 *μ*V in the older group compared to −2.59 *μ*V in the younger group. A significant interaction is reported between age group and time interval for the 2 kHz tone burst (*F* = (3.07,36.86) = 3.73), *P* < 0.05) and for the 4 kHz tone burst (*F* = (3.56,39.33) = 4.11), *P* < 0.01). Results suggest a need to separate mean CAEPs by age group when comparing NH listeners to their HI counterparts in the present study (Figure 2). Overall, NH waveforms can be described as displaying a negative N1 response, followed by clear P2 and N2 responses. No clear P1 response is present for either stimulus condition. The N2 response diminishes in the older age group (reduced amplitude), when compared to the younger age group. 

### 3.4. Aided CAEP Responses and Discussion of HI Case Studies

**Figure 3:**
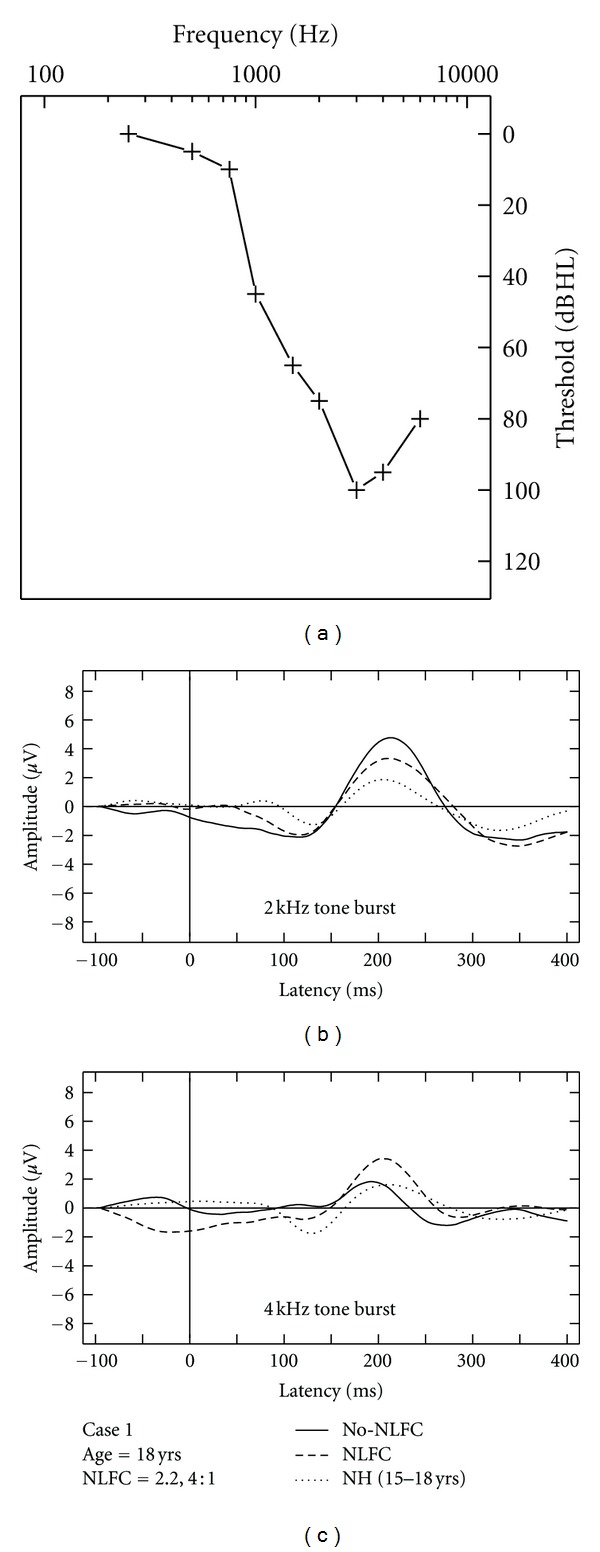
Case-specific grand mean CAEPs displayed across stimulus types and hearing aid conditions: no-NLFC (solid line), NLFC active (dashed line), and for each participant's age-matched NH group (dotted line). Participant age and prescribed NLFC settings (cut-off frequency, compression ratio) are indicated in the legend. The top left corner pane displays test ear hearing thresholds, suspected cochlear dead regions (DR), and responses beyond the audiometric test range.

**Figure 4:**
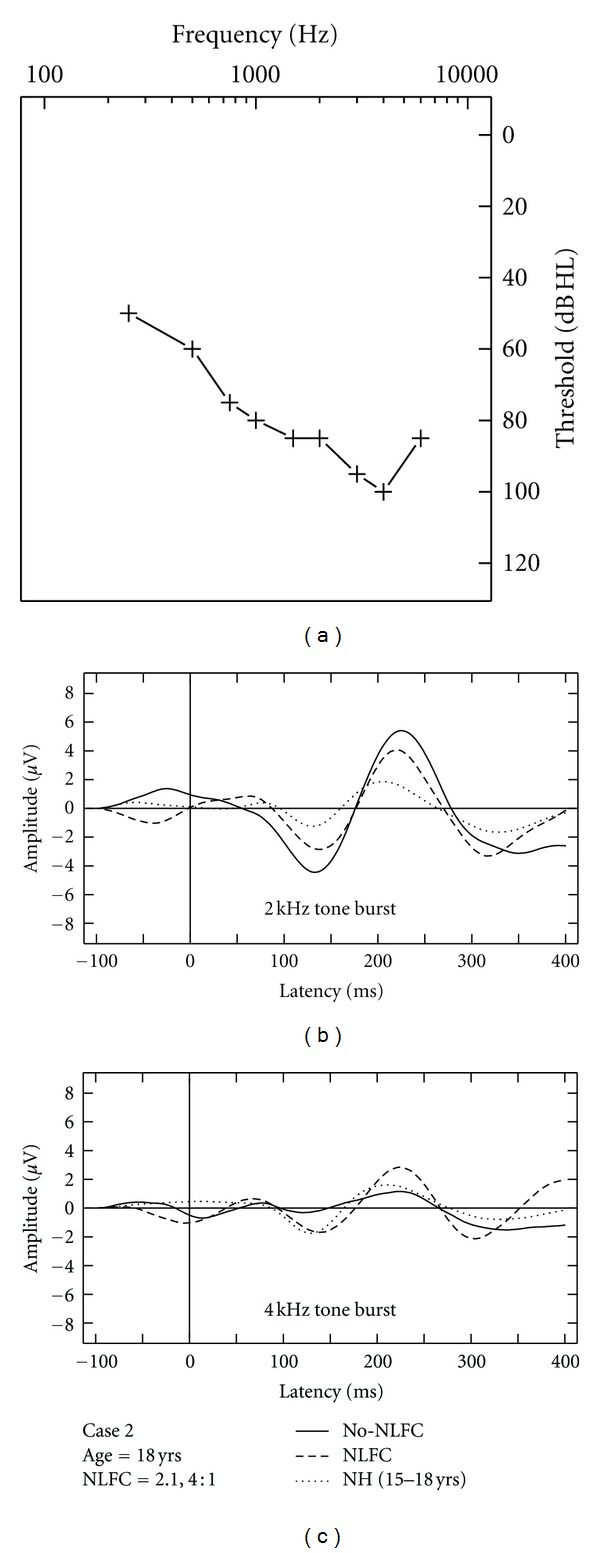
Case-specific grand mean CAEPs displayed across stimulus types and hearing aid conditions: no-NLFC (solid line), NLFC active (dashed line), and for each participant's age-matched NH group (dotted line). Participant age and prescribed NLFC settings (cut-off frequency, compression ratio) are indicated in the legend. The top left corner pane displays test ear hearing thresholds, suspected cochlear dead regions (DR), and responses beyond the audiometric test range.

**Figure 5:**
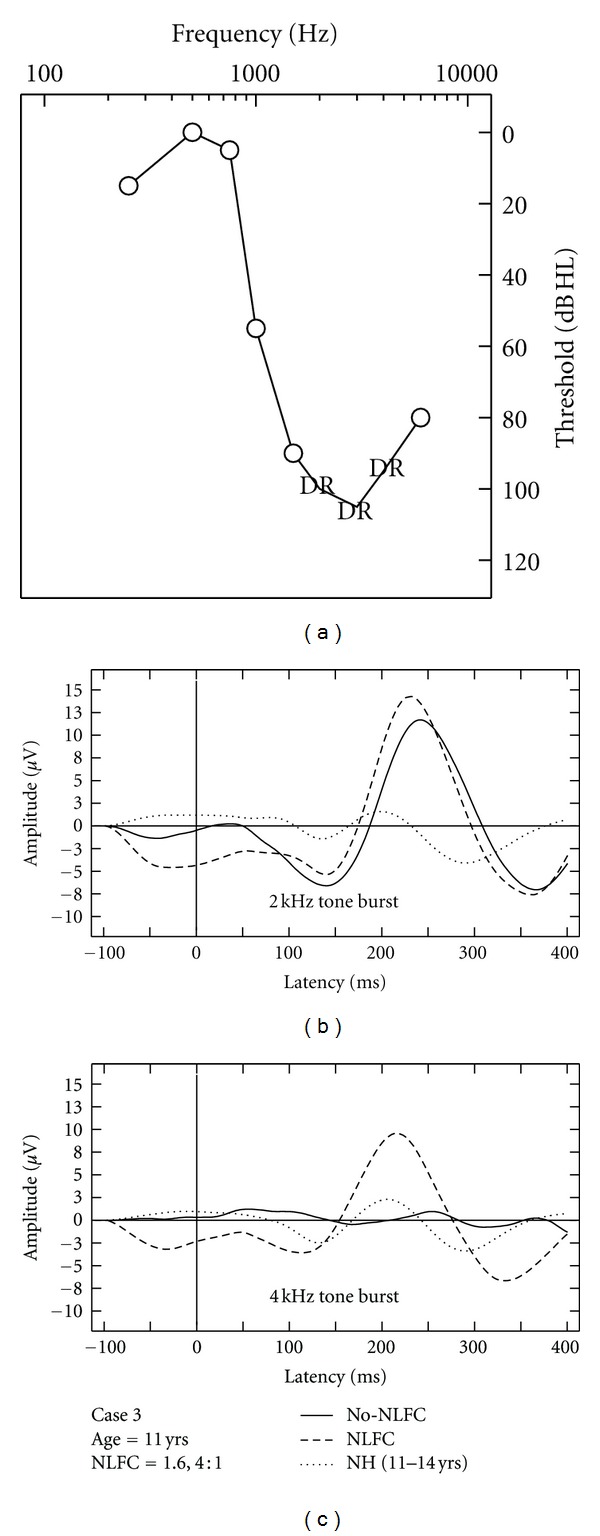
Case-specific grand mean CAEPs displayed across stimulus types and hearing aid conditions: no-NLFC (solid line), NLFC active (dashed line), and for each participant's age-matched NH group (dotted line). Participant age and prescribed NLFC settings (cut-off frequency, compression ratio) are indicated in the legend. The top left corner pane displays test ear hearing thresholds, suspected cochlear dead regions (DR), and responses beyond the audiometric test range.

**Figure 6:**
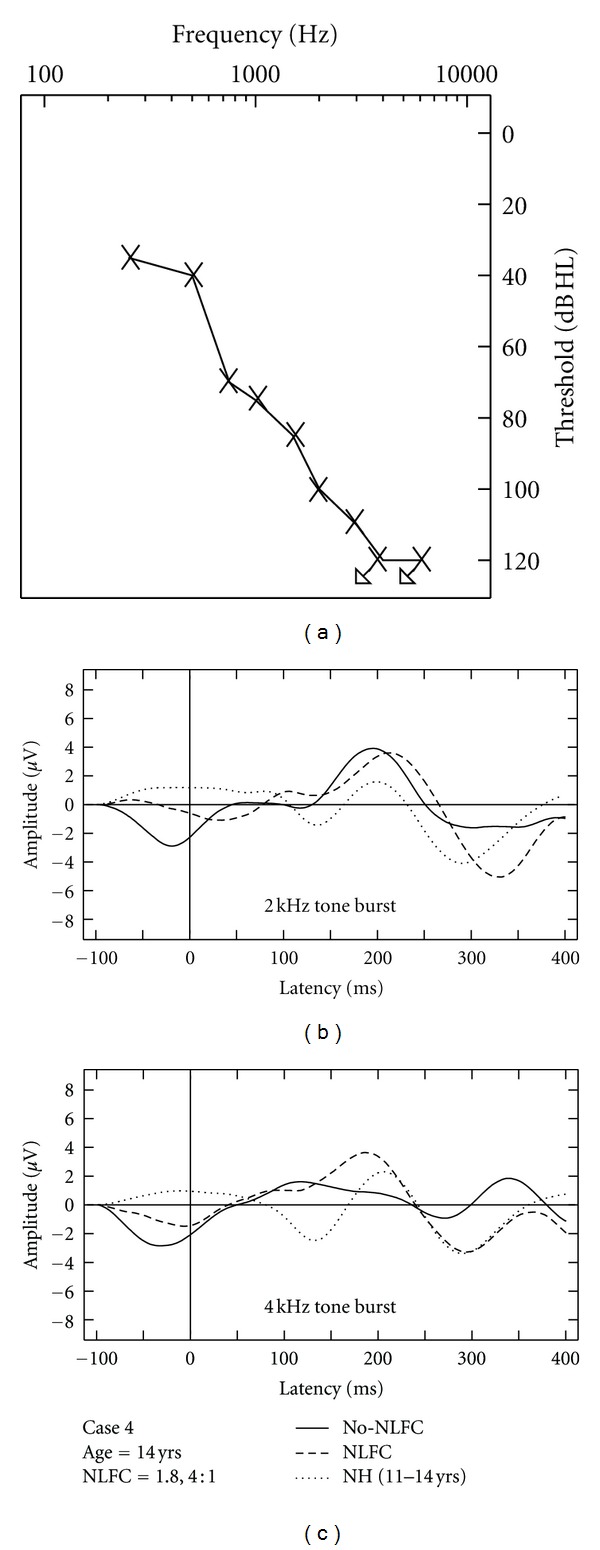
Case-specific grand mean CAEPs displayed across stimulus types and hearing aid conditions: no-NLFC (solid line), NLFC active (dashed line), and for each participant's age-matched NH group (dotted line). Participant age and prescribed NLFC settings (cut-off frequency, compression ratio) are indicated in the legend. The top left corner pane displays test ear hearing thresholds, suspected cochlear dead regions (DR), and responses beyond the audiometric test range.

**Figure 7:**
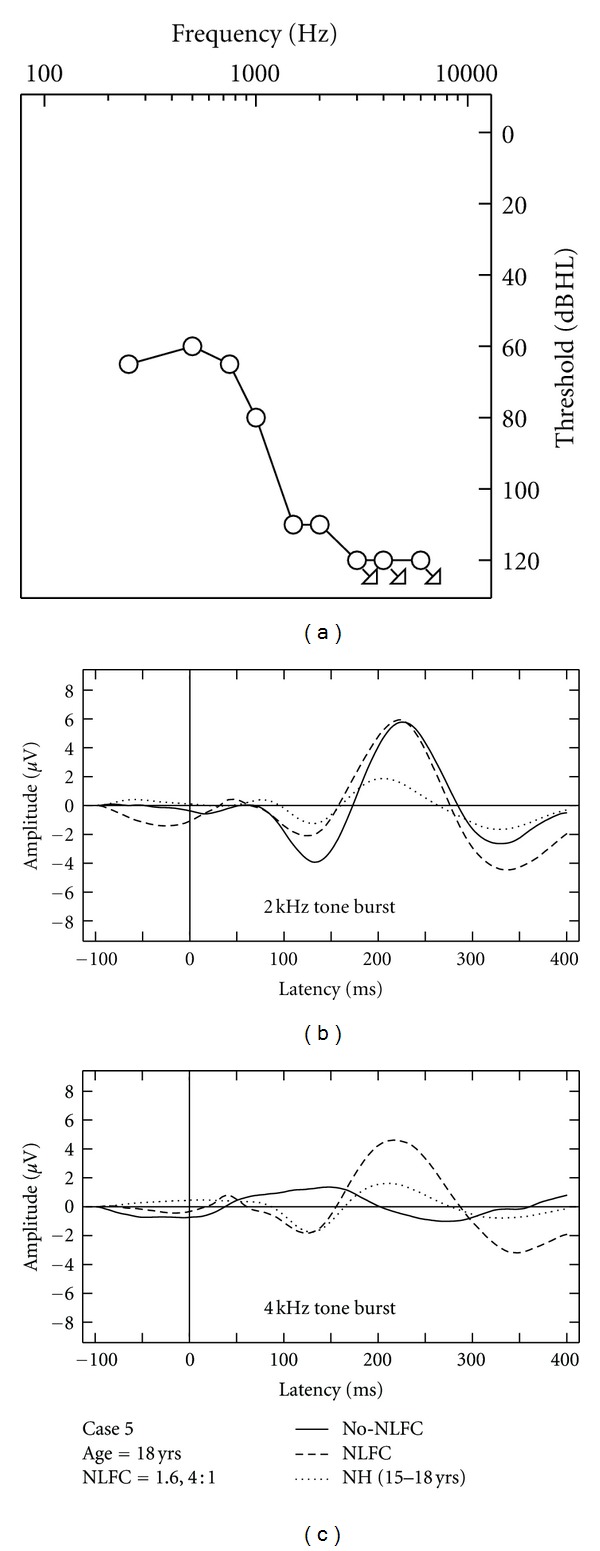
Case-specific grand mean CAEPs displayed across stimulus types and hearing aid conditions: no-NLFC (solid line), NLFC active (dashed line), and for each participant's age-matched NH group (dotted line). Participant age and prescribed NLFC settings (cut-off frequency, compression ratio) are indicated in the legend. The top left corner pane displays test ear hearing thresholds, suspected cochlear dead regions (DR), and responses beyond the audiometric test range.

Due to the small sample of HI listeners included in this study, aided CAEPs are displayed on a case-by-case basis using averaged waveforms across two repetitions, per stimulus and across aided conditions (Figures 3, 4, 5, 6, and 7). Grand mean NH CAEPs are displayed per stimulus (according to matched age group) for comparison purpose. Cases 1 through 5 are displayed in order of greatest to least hearing in the high frequencies, according to HF-PTA values. Case-by-case presentation enables interpretation of change in CAEP findings according to clinical or practical significance [43], considering individual factors such as hearing loss severity, configuration and the use of individualized hearing aid settings. 

Table 1 summarizes waveform interpretation results for all cases, according to stimulus and aided treatment conditions. Present waveforms are reported for the 2 kHz tone burst, with and without NLFC enabled and across all participants. For the 4 kHz tone burst, present waveforms are reported with NLFC enabled across all participants and are judged to be absent for 4 out of the 5 participants, without NLFC enabled. Although waveform morphology appears to be variable across HI cases, some trends are present in the data. Waveforms are primarily dominated by large P2 responses across all aided cases. Many of the aided waveforms appear to have absent P1 responses. Responses measured with the 2 kHz tone burst appear to be larger in amplitude, when compared to those measured with the 4 kHz tone burst, across treatment conditions. 

### 3.5. Comparison of Aided CAEP to Electroacoustic Verification Results

Electroacoustic results suggest that all tone bursts presented in the aided NLFC testing condition and 60 percent of the tone bursts presented in the aided no-NLFC testing condition were made audible by the hearing aid. The latter is a clinically common result for losses of this severity without the use of NLFC and is generally attributable to receiver limitations in modern hearing aids. A summary of these results can also be found in Table 1, along with a description of the corresponding CAEP measure (presence versus absence). For CAEPs judged as present, the estimated SLs ranged between −10 dB and 30.29 dB. For CAEPs judged as absent, the estimated SLs ranged between −18.6 dB and 6.19 dB. These ranges are overlapping but are also consistent with a pattern of higher sensation levels associated with present CAEPs.

## 4. Discussion and Conclusions

This study measured cortical auditory evoked potentials to tone burst stimuli using commercially available clinical testing equipment (Bio-logic Navigator Pro) in 15 NH (unaided) and 5 HI (aided) child listeners (ages 11–18 years). Firstly, this study investigated whether CAEPs could be reliably recorded and interpreted with NH and HI listeners using Bio-logic Navigator Pro testing equipment. Secondly, a case-series approach was used to examine the effects of NLFC hearing aid technology on CAEPs elicited by tone bursts. To facilitate evoked recordings, modifications to the standard testing parameters/equipment were made. In the aided testing conditions, equipment modifications allowed stimuli to be delivered directly to the hearing aid using a DAI hearing aid connector (coupled to an audioshoe). This eliminated the need to control for factors that could have affected the accuracy and reliability of sound-field presentation of stimuli. Due to software limitations, only averaged waveforms could be exported and analyzed offline. 

Waveforms were subjectively interpreted by two experienced examiners using a rating protocol. Literature suggests that that statistical detection of CAEPs is consistent with those of experienced examiners [35, 42]. This study explored the relationship between an objective/statistical index of waveform replicability and subjective interpretation of presence versus absence of CAEPs. The ICC analysis provided a quantitative index of overall similarity of repeated waveforms. This preliminary analysis shows that the ICC, an index assessing test retest reliability, may have the potential to be used to aid subjective interpretation of CAEPs. However, in the absence of a validated pass/fail criterion for ICC values, the ICC data in the present paper merely serve to cross-validate the examiner ratings. Future studies could potentially pursue the development of the ICC as an objective aid to response scoring. Within the NH group, waveforms were judged to be present in most listeners. Waveforms were judged to be absent for one listener with the 4 kHz tone burst and for two listeners for the 2 kHz tone burst. It is unclear why for some NH listeners CAEPs were absent in this study. There have been a few studies that report large differences between CAEP and behavioral thresholds in some participants which imply that CAEPs are not always detected when tested at levels above the behavioral threshold in all participants. Discrepancies of >25 dB between CAEP threshold and behavioral threshold were noted in about 11% of adults tested at 0.5 kHz and 2 kHz [4] and discrepancy of >15 dB in 8.9% of adults at 2 kHz [44]. In HI children (aided and unaided), CAEPs were detected only about 77.8% at 20 dB SL or more [45]. Reasons for this discrepancy may include the specific corrections used to account for short- versus long-duration stimuli, the attention state of the participant and insufficient number of sweeps. It is possible that the results may have been different if recorded using equipment with multiple testing channels and capable of completing online calculations of noise levels. This would allow rejection of eye blinks and other potential sources of noise, as well as monitoring of the level of arousal of the participants. Although none of the participants fell asleep during this study, it is possible that some experienced a reduced level of arousal and consequently had smaller or absent responses. Taken together, these findings from the literature and those from the present student may indicate that interpretation of absent CAEP responses is complex because absent responses may occur even when stimuli are likely suprathreshold. Caution may be indicated when interpreting absent responses in clinical or research contexts.

Waveforms recorded with the NH listeners were found to differ across the age range included in this study. Displayed grand averaged waveforms, separated according to age, suggest that the main source of variability lies within the 250–350 msec range of the response window, with larger N2 amplitudes observed with the younger NH group. These findings are consistent with those reported for a group of normally hearing children listening to click trains: changes in the latency and amplitude values associated with the N2 response were observed up to approximately age 17 [24]. Results from this study differ from the above-mentioned study when considering the presence of observable peaks in the earlier part of the evoked response (specifically the P1 peak). 

Considering the HI listeners, a unique pattern of results was observed across the different cases. The differences observed in waveform morphology may relate to factors including the age range of the participants included in the study (and accompanying developmental effects on waveform morphology) and differences in hearing loss configuration across participants and/or chosen hearing aid settings. In summary, CAEPs were sensitive to changes in audibility across NLFC and no-NLFC hearing aid conditions. Responses appear to be larger, on average, for the 2 kHz tone burst, compared to the 4 kHz tone burst. This is consistent with findings of earlier studies that report an inverse relationship of CAEP amplitude and frequency of tone burst stimuli. The peak-to-peak amplitude was found to decrease as the frequency of the tone bursts increased [46, 47]. 

When comparing NH grand mean waveforms (separated by age group) to the individual averaged waveforms for the HI cases, aided CAEPs tend to be larger in amplitude than the unaided CAEPs measured with the NH group. However, this appears to be the only uniform pattern of results between CAEPs measured with NH and HI participants. Although the ages of the participants were relatively well matched across NH and HI children, the groups were tested with different transducers (broadband insert earphones versus direct audio input to a well-fitted hearing aid). Also, the sensation level of the stimuli was greater for the NH participants than for the HI participants. The HI children in this study had substantial hearing losses, likely accompanied by reduced dynamic ranges of hearing. If we had presented at matched sensation levels across the groups, the stimuli would have been either too loud for the HI participants or very soft for the NH participants. Instead, we presented at middynamic range for all listeners, which in turn results in high sensation levels for NH and low sensation levels for HI participants, respectively. Future studies could potentially consider presenting stimuli at equal loudness by incorporating a loudness model that accounts for the effects of sensorineural hearing loss (e.g., [48]). This may also provide insight into the differences in the relationship between presence of CAEP and SL between the two groups.

Variable CAEP waveform morphology can be observed across the different HI cases when considering gross amplitude and latency differences. For example, present waveforms in Case 3 are larger in amplitude when compared to all other cases. This is also the only case where a suspected cochlear dead region was measured. It is possible that the large response measured with the 2 kHz tone burst reflects overrepresentation of neurons in the cortical regions bordering the cochlear dead region. These findings are closely aligned with those reported in animal model research, describing the use of auditory evoked potential measures to quantify cortical over-representation (e.g., enhanced amplitude responses) corresponding to the frequency region at the cut-off slope of the audiogram in cats [49, 50]. Further research using aided CAEP measures in listeners with steeply sloping audiograms is needed to confirm this speculation. 

All HI participants were long-time hearing aid users who had received a period of time to acclimatize to NLFC prior to beginning testing. The length of time allotted for acclimatization for the purpose of this study is consistent with that reported in the literature (i.e., 12–18 weeks) investigating acclimatization effects in aided speech perception [51, 52]. Presentation levels of tone bursts with higher SLs elicited CAEPs more often than those with lower sensation levels. This was true both with and without the NLFC hearing aid condition. In the cases where NLFC technology improved audibility for a given stimulus, (4 out of the 5 cases, measured with the 4 kHz tone burst), detection of the cortical response also improved. These findings were highly consistent across listeners and are not surprising, given previous work suggesting that the use of hearing aids can improve audibility and thereby increase the probability of eliciting CAEPs the ability to detect CAEPs [7, 8]. A strong relationship between the presence of repeatable aided CAEPs in children, and measures of audibility using electroacoustic verification were reported in this study. This suggests that recording CAEPs to tone burst stimuli, and at a suprathreshold level, can provide physiological evidence that these stimuli have been detected at the level of the auditory cortex with hearing aids fitted. This study did not evaluate specific latency/amplitude differences in the aided P1-N1-P2 complex; rather it generally looked at the presence/absence of a response as indicated by subjective waveform interpretation. Although this study is the first to compare aided CAEPs to electroacoustic verification results, others have compared the relationship between CAEPs and functional outcomes for aided infants [53, 54]. Functional measures of hearing aid performance may provide an important cross-check against aided CAEPs, because both measurement types offer both strengths and limitations.

In summary, this study demonstrated that CAEPs can be recorded with clinical testing equipment in NH and HI children. In the aided testing condition, tone burst stimuli were directly presented to the hearing aid via a DAI connector. Repeatable present waveforms were measured in most participants, although were missing in a small number of NH listeners. Present waveforms in the HI children may have been associated with higher sensation levels. The younger NH children were found to have significantly different responses than the older NH children, with grand averaged waveforms differing mainly between 250 and 350 msec in the response window (the N2 response). For most of the HI cases included in this study, frequency compression hearing aid technology improved audibility of the 4 kHz tone burst; this translated into improved detection of CAEP responses. These findings suggest that the CAEP may be sensitive to the effects of frequency compression signal processing and that frequency compression may have augmented audibility of high-frequency tone bursts on a case-by-case basis. This contribution to the literature provides insight into possible strengths (sensitivity to changes within the aided condition) and limitations (present responses were not always measureable in normal listeners) of CAEP. As this study evaluated *gross* changes in high-frequency audibility, the resulting effects on the CAEP response could be observed as either present or absent. However, further research is needed to assess the effects of hearing aid fine tuning (e.g., changes to hearing aid gain and frequency shaping), or other aspects of hearing aid signal processing, on CAEPs. In addition, more research is needed to validate aided findings reported in this study across a larger group of HI participants including infants and with speech-based stimuli.

## Figures and Tables

**Table 1:** Summary of electroacoustic verification results for all HI cases (1 through 5). Summaries are shown across stimuli (2 kHz and 4 kHz tone bursts) and hearing aid conditions (NLFC enabled and no-NLFC). Results are compared to the corresponding cortical auditory evoked potentials (CAEP) judged as present or absent per condition.

Case	2 kHz NLFC		2 kHz no-NLFC		4 kHz NLFC		4 kHz no-NLFC	
	SL (dB)	CAEP	SL (dB)	CAEP	SL (dB)	CAEP	SL (dB)	CAEP
1	30.29	Present	30.24	Present	11.47	Present	5.36	Present
2	19.36	Present	19.86	Present	10.06	Present	0.39	Absent
3	13.1	Present	13.36	Present	16.29	Present	6.19	Absent
4	9.68	Present	9.66	Present	−7.24	Present	−18.25	Absent
5	0.02	Present	6.51	Present	−10	Present	−18.6	Absent
